# Ambient Air Pollution and Depressive Symptoms in Older Adults: Results from the MOBILIZE Boston Study

**DOI:** 10.1289/ehp.1205909

**Published:** 2014-03-07

**Authors:** Yi Wang, Melissa N. Eliot, Petros Koutrakis, Alexandros Gryparis, Joel D. Schwartz, Brent A. Coull, Murray A. Mittleman, William P. Milberg, Lewis A. Lipsitz, Gregory A. Wellenius

**Affiliations:** 1 Department of Epidemiology, Brown University, Providence, Rhode Island, USA; 2 Department of Environmental Health, and; 3 Department of Biostatistics, Harvard School of Public Health, Boston, Massachusetts, USA; 4 Cardiovascular Epidemiology Research Unit, Beth Israel Deaconess Medical Center, Boston, Massachusetts, USA; 5 Geriatric Neuropsychology Laboratory, Department of Veterans Affairs Medical Center, Boston, Massachusetts, USA; 6 Institute for Aging Research, Hebrew SeniorLife, Boston, Massachusetts, USA, and; 7 Division of Gerontology, Beth Israel Deaconess Medical Center, Boston, Massachusetts, USA

## Abstract

Background: Exposure to ambient air pollution, particularly from traffic, has been associated with adverse cognitive outcomes, but the association with depressive symptoms remains unclear.

Objectives: We investigated the association between exposure to ambient air and traffic pollution and the presence of depressive symptoms among 732 Boston-area adults ≥ 65 years of age (78.1 ± 5.5 years, mean ± SD).

Methods: We assessed depressive symptoms during home interviews using the Revised Center for Epidemiological Studies Depression Scale (CESD-R). We estimated residential distance to the nearest major roadway as a marker of long-term exposure to traffic pollution and assessed short-term exposure to ambient fine particulate matter (PM_2.5_), sulfates, black carbon (BC), ultrafine particles, and gaseous pollutants, averaged over the 2 weeks preceding each assessment. We used generalized estimating equations to estimate the odds ratio (OR) of a CESD-R score ≥ 16 associated with exposure, adjusting for potential confounders. In sensitivity analyses, we considered CESD-R score as a continuous outcome and mean annual residential BC as an alternate marker of long-term exposure to traffic pollution.

Results: We found no evidence of a positive association between depressive symptoms and long-term exposure to traffic pollution or short-term changes in pollutant levels. For example, we found an OR of CESD-R score ≥ 16 of 0.67 (95% CI: 0.46, 0.98) per interquartile range (3.4 μg/m^3^) increase in PM_2.5_ over the 2 weeks preceding assessment.

Conclusions: We found no evidence suggesting that ambient air pollution is associated with depressive symptoms among older adults living in a metropolitan area in attainment of current U.S. regulatory standards.

Citation: Wang Y, Eliot MN, Koutrakis P, Gryparis A, Schwartz JD, Coull BA, Mittleman MA, Milberg WP, Lipsitz LA, Wellenius GA. 2014. Ambient air pollution and depressive symptoms in older adults: results from the MOBILIZE Boston Study. Environ Health Perspect 122:553–558; http://dx.doi.org/10.1289/ehp.1205909

## Introduction

Depressive symptoms are highly prevalent in older adults (Thielke et al. 2010) and are an important risk factor for cardiovascular morbidity and mortality. Specifically, high levels of depressive symptoms have been associated with higher risk of all-cause and cardiovascular death, coronary heart disease events, and stroke (Arbelaez et al. 2007; Glymour et al. 2010; Schulz et al. 2000; Whooley et al. 2008); progression of coronary artery atherosclerosis (Janssen et al. 2011); higher health care utilization and costs (Unutzer et al. 1997); and decreased quality of life (Ruo et al. 2003).

In older adults, long-term exposure to higher levels of ambient fine particulate matter (PM with aerodynamic diameter ≤ 2.5 μm; PM_2.5_) and traffic pollution has been associated with poorer cognitive function (Power et al. 2011; Ranft et al. 2009; Wellenius et al. 2012a) and an increased rate of cognitive decline (Weuve et al. 2012). Exposure to ambient air pollution may also plausibly increase the risk of depressive symptoms. Specifically, separate lines of evidence suggest that *a*) air pollution can cause systemic inflammation, neuroinflammation, oxidative stress, cerebrovascular damage, and neurodegenerative pathology (reviewed by Block and Calderón-Garcidueñas 2009), and *b*) inflammation and vascular disease contribute to the risk of, or exacerbate, specific types of depression (Anisman and Hayley 2012; Dantzer et al. 2008; Krishnadas and Cavanagh 2012; Sneed and Culang-Reinlieb 2011). Thus, exposure to specific pollutants or pollution sources might increase the risk of depressive symptoms by promoting neuroinflammation and/or cerebrovascular injury. Although exposure to secondhand tobacco smoke has been associated with the increased prevalence of depressive symptoms in children and adolescents (Bandiera et al. 2011), the association between long-term exposure to ambient air pollution and depressive symptoms has not been previously evaluated. In addition, daily fluctuations in ambient levels of particles with aerodynamic diameter ≤ 10 μm (PM_10_), ozone (O_3_), and nitrogen dioxide (NO_2_) have been associated with more severe depressive symptoms among older adult participants (Lim et al. 2012) as well as a higher rate of emergency department visits for depression and attempted suicide (Kim et al. 2010; Szyszkowicz 2007; Szyszkowicz et al. 2009, 2010), although results from these studies were not entirely consistent.

The goal of the present study was to evaluate the association of both long-term exposure to traffic pollution and short-term exposure to ambient air pollution with the presence of depressive symptoms in a prospective cohort study of community-dwelling older adults.

## Materials and Methods

The MOBILIZE Boston Study is a prospective study of novel risk factors for falls in adults ≥ 65 years of age (Leveille et al. 2008). Briefly, between 2005 and 2008, we recruited 765 community-dwelling men and women able to communicate in English and walk 20 feet without assistance and residing within a 5-mile radius of the study clinic. Individuals not planning to reside in the study area for ≥ 2 years, those with severe vision or hearing impairment, and those with cognitive impairment [defined as a Mini-Mental State Examination (MMSE) score of < 18] were not eligible to participate. Participant recruitment was based on a simple random sample of persons on town lists within the study area. Upon enrollment, participants completed an in-home interview followed within 4 weeks by an in-clinic evaluation. A second assessment consisting of an in-home interview and clinic examination was performed 12–18 months after the baseline assessment. All participants provided written informed consent, and this analysis was approved by the institutional review boards at Hebrew SeniorLife and Brown University.

We assessed depressive symptoms during each home visit using the 20-item Revised Center for Epidemiological Studies Depression Scale (CESD-R) (Eaton et al. 2004; Van Dam and Earleywine 2011). Trained staff read each question out loud to participants and recorded participant answers. The CESD-R is a validated instrument for assessing the presence of depressive symptoms over the preceding 2 weeks in community and epidemiologic studies. CESD-R scores were recalibrated to range from 0 to 60, as previously described (Eaton et al. 2004), with higher scores reflecting the presence of more symptoms of depression. We dichotomized the revised CESD-R score using a cut point of ≥ 16, as is common practice when using the original Center for Epidemiological Studies Depression Scale (CES-D) (Smarr and Keefer 2011).

During the home visit, we also obtained detailed information on participant age, race/ethnicity, sex, education, household income, medical history, current medications, physical activity, usual alcohol consumption, and smoking history, as previously described (Leveille et al. 2008). We used the Physical Activity Scale for the Elderly (PASE), a validated 10-item instrument for assessing physical activity in epidemiological studies of older adults (Washburn et al. 1993). Smoking history was classified as never, past, or current smokers.

During each clinic examination we measured height and weight, collected a non-fasting venous blood sample, and measured supine blood pressure, as previously described (Wellenius et al. 2012c). Participants were classified as normotensive if their blood pressure was < 140/90 mmHg, they had no history of hypertension, and they were not receiving medications prescribed for hypertension; controlled hypertensive if their blood pressure was < 140/90 mmHg and they had a history of hypertension or were receiving antihypertensive medication; and uncontrolled hypertensive if their blood pressure was ≥ 140/90 mmHg. Participants were classified as having diabetes mellitus if they reported a past diagnosis of diabetes, reported using any diabetes medications, had measured hemoglobin A1c levels ≥ 7%, or had a random glucose measurement ≥ 200 mg/dL; and as having hyperlipidemia if their total cholesterol was ≥ 200 mg/dL, their low-density lipoprotein cholesterol level was ≥ 130 mg/dL, or they reported taking lipid-lowering medications.

*Exposure assessment*. We calculated residential distance to the nearest major roadway as a marker of long-term exposure to traffic pollution. We used ArcGIS (version 9.2; ESRI Inc., Redlands, CA) to geocode participants’ addresses and calculate the Euclidean distance from residence to the nearest major roadway, defined as a road with U.S. Census Feature Class Code A1 (primary highway with limited access) or A2 (primary road without limited access), as previously described (Wellenius et al. 2012a). In a secondary analysis, we estimated daily outdoor black carbon (BC) levels (a marker of traffic pollution) at each participant’s residential address using a validated spatial-temporal land-use regression model, as previously described (Gryparis et al. 2007). We averaged estimated residential BC over the 365 days preceding each participant’s assessment to create a metric of long-term exposure to BC.

We measured ambient levels of PM_2.5_, BC, ultrafine particles (UFP), and sulfates (SO_4_^2–^) continuously at the Harvard School of Public Health stationary ambient monitoring site and derived daily averages, as previously described (Kang et al. 2010; Zanobetti and Schwartz 2006). The monitoring site was located < 20 km from the home of any study participant. We calculated daily concentrations of gaseous pollutants including O_3_, carbon monoxide (CO), nitrogen monoxide (NO), and NO_2_ based on hourly data obtained from the Massachusetts Department of Environmental Protection monitoring sites. We calculated daily ambient and dew point temperatures and barometric pressure based on hourly data obtained from the National Weather Service station at Boston Logan Airport (Boston, MA). For each pollutant, we calculated moving averages of 1, 2, 3, 5, 7, and 14 days preceding the day of CESD-R assessment as the metric for short-term exposure to ambient air pollution. These moving averages were selected *a priori* based on eariler publications (Lim et al. 2012; Szyszkowicz 2007; Szyszkowicz et al. 2009, 2010) and on the basis that the CESD-R evaluates depressive symptoms within the preceding 14 days.

*Statistical analyses*. After excluding 31 participants who did not complete the CESD-R survey and data from two visits that fell on weekends, data from 1,314 baseline and follow-up assessments of 732 participants were available for the present analysis. Internal consistency of the CESD-R in this study was good (Cronbach’s α = 0.84) and comparable to what has been previously reported (Van Dam and Earleywine 2011).

We used generalized estimating equations with a logit link function and an exchangeable correlation matrix to assess the association between long-term exposure to traffic pollution and the presence of depressive symptoms. Specifically, we estimated the log odds of having a CESD-R score ≥ 16 associated with differences in residential distance to the nearest major roadway while accounting for repeated assessments within participants. In all models, we adjusted for potential confounding by age [natural spline with 3 degrees of freedom (df)], sex, race/ethnicity (non-Hispanic white vs. other), visit (baseline vs. follow-up), season (four categories), day of week (four indicator variables), household income (below vs. above median), education (no college degree, 4-year college or vocational school, graduate school) and neighborhood socioeconomic status (linear continuous) using two census tract–level variables: *a*) percent of population that is not non-Hispanic white, and *b*) percent of population with college degree or above. In a second model, we additionally adjusted for baseline body mass index (BMI; natural spline with 3 df), physical activity (linear continuous), alcohol consumption (linear continuous), smoking, diabetes mellitus, hypertension (normotension, controlled hypertension, uncontrolled hypertension), hyperlipidermia, and use of antidepressant medication. These additional variables may be confounders, but they could also be intermediates along the causal path from traffic pollution to depressive symptoms. We modeled residential distance to the nearest major roadway with five categories (< 100, 100–250, 250–500, 500–1,000, > 1,000 m) and tested for linear trend by assigning the median distance to each category and including the term as a continuous variable in regression models.

We performed additional sensitivity analyses to evaluate the robustness of our findings. First, we considered residential distance to nearest major roadway as a continuous variable on both the original scale and after taking the natural logarithm. Second, we considered annual mean residential BC levels instead of residential distance to major roadway as a marker of long-term exposure to traffic pollution. Third, we defined depressive symptoms as being present if either CESD-R score ≥ 16 or participants reported taking antidepressant medication. Fourth, we used linear mixed effects models to model CESD-R score as a continuous variable rather than a dichotomous outcome. Fifth, we restricted analyses to participants with an MMSE score > 24 to minimize the opportunity for bias from participants with evidence of more subtle cognitive impairment.

We used a similar modeling strategy to evaluate the association between mean ambient air pollution levels in the preceding 2 weeks and the presence of depressive symptoms. In these models we adjusted for potential confounding by age, sex, race/ethnicity, visit, ambient and dew point temperatures, barometric pressure, day of week, season (four indicator variables for season and sine and cosine of calendar day), and long-term temporal trends (calendar day as a linear continuous variable). Ambient temperature, dew point temperature, and barometric pressure were each modeled with natural cubic splines with 3 df. In sensitivity analyses we additionally adjusted for BMI, physical activity, alcohol consumption, smoking, diabetes mellitus, hypertension, hyperlipidermia, and use of antidepressant medication; all modeled as described above. We examined the results for the entire year and repeated the analyses restricted to measurements from visits occurring in either the warm (April–September) or cool (October–March) seasons.

We performed all analyses using *R* statistical software (R version 2.13; http://www.r-project.org/foundation/). A two-sided *p*-value of < 0.05 was considered statistically significant.

## Results

At baseline, MOBILIZE Boston Study participants were predominantly non-Hispanic white and female, with a mean age of 78.1 years (SD = 5.5) (Table 1). The presence of depressive symptoms was assessed twice in 582 of 732 (79%) of the participants, a median of 16.0 months apart, and only once in the remaining participants. CESD-R scores ranged from 0 to 45 with a median of 3. The 62 (8.5%) participants with a CESD-R score ≥ 16 at baseline were less likely to be non-Hispanic white (62.9% vs. 79.3%), have a college degree (48.4% vs. 67.6%), or be as physically active, and more likely to be taking antidepressant medication (30.6% vs. 10.9%) than other participants.

**Table 1:** Baseline characteristics of 732 participants from the MOBILIZE Boston Study according to residential distance to a major roadway.

Characteristic	All (*n* = 732)^*a*^	< 100 m (*n* = 74)	100–250 m (*n* = 78)	250–500 m (*n* = 130)	500–1,000 m (*n* = 213)	> 1,000 m (*n* = 228)
Age (years; mean ± SD)	78.1 ± 5.5	78.5 ± 5.0	77.5 ± 4.8	78.3 ± 5.5	78.3 ± 5.7	77.8 ± 5.6
Male (%)	35.8	37.8	26.9	35.4	36.6	38.2
Non-Hispanic white (%)	77.7	78.4	74.4	79.2	76.5	78.9
Education (%)
High school or less	33.9	28.4	26.9	41.5	32.9	34.6
College or vocational school	34.8	45.9	38.5	34.6	30.5	33.8
Graduate school	31.1	24.3	34.6	23.8	36.6	31.6
Household income ≤ $35,000 (%)	55.1	60.8	60.3	60.8	50.7	51.8
BMI (kg/m^3^; mean ± SD)	27.3 ± 5.1	27.5 ± 4.5	27.7 ± 5.0	26.9 ± 4.6	27.1 ± 5.4	27.6 ± 5.3
Alcohol consumption (drinks/day; mean ± SD)	0.9 ± 1.0	0.7 ± 0.8	1.0 ± 0.7	0.9 ± 0.9	1.0 ± 1.2	1.0 ± 0.9
Ever smoker (%)						
Never	42.9	36.5	42.3	41.5	44.1	45.6
Past	52.2	54.1	60.3	53.8	52.6	49.1
Current	4.8	9.4	5.1	4.6	2.8	5.3
Hypertension (%)	78.0	75.7	73.1	85.4	75.6	78.9
Diabetes mellitus (%)	20.1	25.7	5.1	24.6	21.6	18.9
Hyperlipidemia (%)	47.1	54.1	42.3	40.8	50.2	46.5
MMSE (mean ± SD)	27.2 ± 2.6	27.0 ± 2.6	26.9 ± 2.7	27.0 ± 2.6	27.2 ± 2.7	27.3 ± 2.5
CESD-R ≥ 16 (%)	8.5	6.8	11.5	14.6	7.0	6.1
Antidepressant use (%)	12.4	13.5	24.4	13.1	9.4	10.5
CESD-R assessed at both visits (%)	79.5	78.4	79.5	76.9	79.3	81.6
^***a***^Percent of missing data: BMI (2.5%), hypertension (1.1%), alcohol consumption (0.1%), education (0.1%), smoking (0.1%), antidepressant use (2.7%)						

*Long-term exposure to traffic pollution*. Residential distance to the nearest major roadway varied from near 0 m to just over 3 km, with a median distance of 667 m and 10% of participants (*n* = 74) living within 100 m of a major roadway (Table 1). Participant characteristics varied across categories of residential distance to nearest major roadway, but not in a strictly monotonic manner. For example, participants living 100–250 m from a major roadway had the lowest mean age, were most likely to be female, were the least likely to be non-Hispanic white and have diabetes or hypertension, and were most likely to report use of antidepressant medication (Table 1). Annual residential BC exposure was approximately normally distributed with a mean of 0.37 μg/m^3^ and a SD of 0.12 μg/m^3^. The Pearson correlation between residential proximity to major roadway and annual residential BC was 0.11.

We did not find consistent evidence of an association between categories of residential distance to the nearest major roadway and the presence of depressive symptoms, with a negative (although not statistically significant) association observed for those living closest to roadways and elevated (but not statistically significant) associations observed for those living 100–500 m from a major roadway (Table 2). In sensitivity analyses considering mean annual residential BC concentrations as the marker of long-term exposure to traffic pollution, we similarly found no evidence of increased presence of depressive symptoms per interquartile range (IQR) (0.11 μg/m^3^) difference in mean annual residential BC concentration (see Supplemental Material, Table S1). Results were qualitatively similar in additional sensitivity analyses considering residential distance to roadway as a continuous variable on both the original scale and after taking the natural logarithm (data not shown), considering the presence of depressive symptoms defined as either a CESD-R score ≥ 16 or reported use of antidepressant medication (resulting in an additional 61 participants being identified as having depressive symptoms; results not shown), considering CESD-R score as a linear continuous variable (Table 2; see also Supplemental Material, Table S1), or restricted to those participants with an MMSE > 24 (resulting in 21 fewer participants with CESD-R ≥ 16; results not shown).

**Table 2:** Association [OR (95% CI)] between categories of residential distance to nearest major roadway and the presence of depressive symptoms among 732 participants from the MOBILIZE Boston Study.

Outcome and model	< 100 m	100–250 m	250–500 m	500–1,000 m	> 1,000 m	*p*_trend_
CESD-R ≥ 16 vs. < 16
Model 1^*a*^	0.81 (0.33, 2.01)	1.68 (0.82, 3.44)	1.58 (0.85, 2.95)	0.91 (0.51, 1.64)	1.0 (Referent)	0.27
Model 2^*b*^	0.63 (0.26, 1.57)	1.45 (0.68, 3.11)	1.66 (0.86, 3.17)	0.94 (0.51, 1.74)	1.0 (Referent)	0.46
CESD-R as a continuous variable
Model 1^*a*^	–0.73 (–2.44,0.99)	0.81 (–0.86,2.48)	0.30 (–1.10, 1.71)	–0.19 (–1.40, 1.03)	1.0 (Referent)	0.87
Model 2^*b*^	–1.16 (–2.86, 0.54)	0.27 (–1.36, 1.90)	0.23 (–1.14, 1.60)	–0.06 (–1.24, 1.12)	1.0 (Referent)	0.69
Estimates represent ORs (95% CIs) for CESD-R ≥ 16 as a dichotomous outcome, and the absolute difference in CESD-R score (95% CI) for CESD-R score modeled as a continuous outcome. ^***a***^Adjusted for age, sex, race/ethnicity, visit, season, day of week, household income, education, and neighborhood socioeconomic status. ^***b***^Additionally adjusted for BMI, physical activity, alcohol consumption, smoking, diabetes mellitus, hypertension, hyperlipidemia, and use of antidepressant medication.						

*Short-term changes in ambient pollution levels*. The Boston metropolitan area was in compliance of the U.S. National Ambient Air Quality Standards for the duration of the study period and pollutant levels were lower than in many other major cities (Table 3). Pollutant levels were moderately to strongly positively correlated with each other, with the exception of O_3_, which was uncorrelated with CO and NO_2_ and negatively correlated with other pollutants (see Supplemental Material, Table S2).

**Table 3:** Average daily levels of ambient air pollutants, overall and stratified by season.^*a*^

Pollutant	Unit	All year		Warm season		Cool season	
		Mean ± SD	Median	Mean ± SD	Median	Mean ± SD	Median
PM_2.5_	μg/m^3^	8.6 ± 4.9	7.3	9.1 ± 5.6	7.4	8.2 ± 4.2	7.1
SO_4_^2–^	μg/m^3^	2.6 ± 2.1	2.0	2.9 ± 2.6	2.0	2.3 ± 1.4	2.0
BC	μg/m^3^	0.62 ± 0.35	0.55	0.66 ± 0.33	0.62	0.60 ± 0.36	0.52
UFP	1,000/m^3^	15.3 ± 6.7	14.5	11.7 ± 4.5	10.6	18.2 ± 6.8	17.1
O_3_	ppb	24.5 ± 10.8	23.4	30.0 ± 10.7	29.1	19.4 ± 8.1	18.8
CO	ppm	0.31 ± 0.14	0.30	0.30 ± 0.12	0.29	0.32 ± 0.16	0.30
NO	ppb	13.2 ± 10.0	10.2	9.2 ± 4.4	8.3	16.8 ± 12.2	13.2
NO_2_	ppb	16.7 ± 5.6	15.9	14.7 ± 4.2	14.4	18.6 ± 6.1	18.1
^***a***^Warm season: April–September, cool season: October–March.							

We found no evidence of a positive association between mean pollutant levels over the 2 weeks preceding CESD-R administration and presence of depressive symptoms (Table 4), although a statistically significant negative association was observed with PM_2.5_ (OR = 0.67; 95% CI: 0.46, 0.98 per 3.4 μg/m^3^ increase). We found no consistent evidence of an association between the presence of depressive symptoms and mean pollutant levels averaged over shorter periods preceding assessment (data not shown). Additional adjustment for BMI, physical activity, alcohol consumption, smoking, diabetes mellitus, hypertension, hyperlipidermia, and use of antidepressant medication did not materially alter the results (data not shown). Results were qualitatively similar in sensitivity analyses considering the presence of depressive symptoms defined as either a CESD-R score ≥ 16 or reported use of antidepressant medication (data not shown), considering CESD-R score as a continuous variable, or restricted to those participants with an MMSE > 24 (data not shown). The results were not materially different in analyses stratified by season, whether effect estimates were scaled to season-specific IQRs (see Supplemental Material, Table S3) or all-year IQRs (data not shown).

**Table 4:** Odds ratios (ORs) of CESD-R ≥ 16 associated with an interquartile range (IQR) difference in pollutant levels over the preceding 2 weeks.^*a*^

Pollutant	Unit	IQR	OR (95% CI)	*p-*Value
PM_2.5_	μg/m^3^	3.40	0.67 (0.46,0.98)	0.037
SO_4_^2–^	μg/m^3^	1.10	0.89 (0.65,1.22)	0.48
BC	μg/m^3^	0.23	1.00 (0.75,1.33)	0.98
UFP	1,000/m^3^	6.63	1.04 (0.68,1.57)	0.87
O_3_	ppb	13.45	0.71 (0.46,1.09)	0.12
CO	ppm	0.12	1.14 (0.90,1.44)	0.28
NO	ppb	8.20	1.30 (0.96, 1.77)	0.092
NO_2_	ppb	4.07	1.32 (0.99,1.76)	0.055
^***a***^Models adjusted for age, sex, race/ethnicity, visit, ambient and dew point temperatures, barometric pressure, day of week, season, and long-term temporal trends.				

## Discussion

In this cohort of community-dwelling older adults, the presence of depressive symptoms within the preceding 2 weeks, as assessed by the CESD-R, was not associated with markers of long-term exposure to traffic pollution. We also found no evidence suggestive of a positive association between depressive symptoms and mean ambient pollutant levels in the preceding 2 weeks. These results were robust to a number of sensitivity analyses.

We are not aware of any earlier studies evaluating the association between long-term exposure to traffic pollution and depressive symptoms in humans. However, one toxicologic study reported that mice exposed to PM_2.5_ for 10 months were more likely to exhibit depressive-like responses compared to control animals (Fonken et al. 2011). Given the difference in species and that PM_2.5_ levels in that study were an order of magnitude higher than the ambient levels observed in the present study, the relevance of the results from that toxicological study is unclear.

Our results regarding the association between short-term changes in ambient levels of particulate matter and gaseous pollutants differ from the results of a recent study with repeated assessment of depressive symptoms using the Korean 15-item Short Geriatric Depression Scale (SGDS) in 547 community-dwelling elderly persons (mean ± SD, 71 ± 5 years of age) in Seoul, Korea (Lim et al. 2012). That study found a 17.0% (95% CI: 4.9, 30.5%) higher score associated with a 24-μg/m^3^ increase in the 3-day moving average of PM_10_, a 43.7% (95% CI: 11.5, 85.2%) higher score per 15-ppb increase in the 3-day moving average of O_3_, and a 32.8% (95% CI: 12.6, 56.6%) higher score per 37-ppb increase in the 7-day moving average of NO_2_. Although both the SGDS and CESD-R are screening tools for depressive symptoms, the two instruments likely measure somewhat different aspects or components of depressive symptoms (Gerety et al. 1994; Smarr and Keefer 2011). Alternatively, or in addition, differences in the sources, composition and relative concentrations of pollutants, or differences in participant characteristics may also contribute to the divergent results. For example, levels of O_3_, CO, and NO_2_ in the study by Lim et al. (2012) were more than two times higher than those observed in the present study. In addition, Lim et al (2012) evaluated the association with PM_10_, whereas we considered PM_2.5_. However, there is a large body of literature establishing the association between present-day Boston-area air pollution levels and increased risk of cardiovascular and cerebrovascular events and subtle shifts in cardiovascular physiology (e.g., O’Neill et al. 2005; Wellenius et al. 2012b, 2013; Zanobetti et al. 2004). Nonetheless, it is plausible that higher mean levels or larger changes in daily levels would have been needed for us to detect changes in CESD-R scores among MOBILIZE Boston Study participants.

Szyszkowicz (2007) previously reported associations between PM_10_, (but not PM_2.5_), CO, and NO_2_ and the rate of same-day emergency department visits with a discharge diagnosis of depression in Edmonton, Canada, which has pollution levels similar to Boston’s. The associations were generally stronger or only observed in the warm months of the year and/or only among women. A subsequent analysis across seven Canadian cities (including the previous data from Edmonton) showed similar associations when both sexes were considered jointly (Szyszkowicz et al. 2009). Szyszkowicz et al. (2010) found some evidence of associations between emergency department visits for suicide attempts in Vancouver and PM_10_, CO, and NO_2_ levels, but mostly in subanalyses stratified by season and sex, whereas Kim et al. (2010) reported associations between deaths due to suicide and daily PM_10_ and PM_2.5_ in Korea. These early reports raise the intriguing possibility that depression episodes may be associated with recent levels of ambient air pollutants. Our study does not directly address this hypothesis because the CESD-R assesses the presence of depressive symptoms within the preceding 2 weeks rather than depression episodes or the presence of clinical depression.

Our study has some limitations. First, the prevalence of depressive symptoms in our study was low (8.4%), close to the lower limit of the spectrum reported for community-dwelling older adults in the United States (Thielke et al. 2010). This limited the statistical power of our study and possibly limits the generalizability of our results to other older adult populations. Moreover, there were very few participants with CESD-R scores consistent with a probable depressive case as defined by Van Dam and Earleywine (2011), precluding our use of this outcome. Second, because we cannot exclude the possibility that participation in the MOBILIZE Boston Study was also related to residential proximity to major roadways, our results may reflect some amount of selection bias. Third, our analysis of the association with short-term changes in ambient pollutants was based on pollutant levels measured at a single monitoring site, potentially resulting in some exposure misclassification. However, all participants resided within 20 km of the study site, a distance over which PM_2.5_ levels measured at the monitoring site have been shown to be strong proxies for personal exposure to particles of ambient origin (Brown et al. 2009). Although exposure misclassification may have increased the width of our CIs, it was not expected to have biased our health effect estimates in either direction (Zeger et al. 2000). In addition, we did not have information on the residential histories of participants before their study enrollment, potentially leading to some exposure misclassification in our analysis of the association with long-term exposure to traffic pollution. However, residential mobility in this age group is expected to be low (Bradley and Longino 2009). Fourth, we do not have information on the amount of time participants spent outdoors, potentially leading to additional exposure misclassification. Finally, the results may not be generalizable to other geographic locations with different population characteristics, pollution sources, and meteorological factors.

On the other hand, strengths of this study include a relatively large, prospective cohort study of well-characterized community-dwelling older adults evaluated repeatedly for depressive symptoms. In addition, at baseline MOBILIZE Boston Study participants were largely representative of older adults in the Boston area in terms of age, sex, race, and ethnicity (Leveille et al. 2008), enhancing the generalizability of our findings.

## Conclusion

In this relatively large, prospective cohort study of community-dwelling older adults, we found no evidence suggestive of a positive association between the presence of depressive symptoms and markers of long-term exposure to traffic pollution. In addition, short-term changes in levels of ambient air pollutants were also not associated with the presence of depressive symptoms. Although we cannot exclude the possibility of a very small effect, these results do not support the notion that short-term or long-term exposure to ambient air pollutants are positively associated with depressive symptoms in older adults living in a metropolitan area in attainment of current U.S. regulatory standards.

## Supplemental Material

(181 KB) PDF
