# Supplementary material for: Ambient Air Pollution and Depressive Symptoms in Older Adults: Results from the MOBILIZE Boston Study
Source: Environ Health Perspect. 2014 Mar 7;122(6):553–8. doi: 10.1289/ehp.1205909 (PMC4050499; doi:10.1289/ehp.1205909)
Supplement: (181 KB) PDF [file ehp.1205909.s001.pdf]

Environmental Health Perspectives doi:10.1289/ehp.1205909

Supplemental Material

Ambient Air Pollution and Depressive Symptoms in Older Adults: Results from the MOBILIZE Boston Study

Yi Wang, Melissa N. Eliot, Petros Koutrakis, Alexandros Gryparis, Joel D. Schwartz, Brent A. Coull, Murray A. Mittleman, William P. Milberg, Lewis A. Lipsitz, and Gregory A. Wellenius

Table S1. Association between mean annual residential black carbon and the presence of depressive symptoms among 732 participants of the MOBILIZE Boston Study. Estimates represent odds ratios (95% CI) for CESD-R ≥ 16 as a dichotomous outcome, and the absolute difference in CESD-R score (95% CI) for CESD-R score modeled as a continuous outcome associated with an interquartile range (0.11 μg/m^3) increase in mean annual residential black carbon concentration.

**Outcome and Model**	**Point estimate (95% CI)**	
**CESD-R ≥ 16 vs < 16**		**P-value**
Model 1[a]	0.92 (0.72,1.19)	0.52
Model 2[b]	0.86 (0.67,1.11)	0.25
**CESD-R as a continuous variable**		**P-value**
Model 1[a]	0.05 (-0.43,0.52)	0.84
Model 2[b]	0.03 (-0.44,0.48)	0.91

[a]Adjusted for age, sex, race/ethnicity, visit, season, day of week, household income, education and neighborhood socioeconomic status at baseline. [b]Additionally adjusted for body mass index, physical activity, alcohol consumption, smoking, diabetes mellitus, hypertension, and hyperlipidermia and use of antidepressant medication at baseline.

Table S2. Spearman correlation matrix for daily average pollutant levels in Boston, 2005-2008.

Pollutant	$PM_{2.5}$	SO_4^{2-}	BC	UFP	O_3	CO	NO	NO_2
$PM_{2.5}$		0.87	0.61	-0.27	0.54	0.18	-0.20	0.29
SO_4^{2-}	0.83		0.54	-0.34	0.51	0.20	-0.28	0.17
BC	0.77	0.65		-0.01	0.05	0.36	0.31	0.63
UFP	0.12	-0.01	0.17		-0.25	0.12	0.41	0.36
O_3	-0.38	-0.23	-0.53	-0.27		0.05	-0.53	0.02
CO	0.49	0.44	0.59	0.20	-0.24		0.12	0.33
NO	0.60	0.42	0.78	0.33	-0.67	0.58		0.54
NO_2	0.65	0.53	0.67	0.34	-0.52	0.53	0.70	

Correlations in warm season are shown in the upper right triangle and correlations in the cool season are shown in the lower left triangle. Symbols: BC = black carbon; UFP = ultrafine particles.

Table S3. Odds ratios (ORs) of CESD-R ≥ 16 associated with an interquartile range (IQR) increase in pollutant levels over the past 2 weeks in the warm and cool seasons.[a] Note that IQR for each pollutant is season-specific.

Pollutant	Unit	Warm Season IQR	Warm Season OR (95% CI)	p	Cool Season IQR	Cool Season OR (95% CI)	p
$PM_{2.5}$	µg/m^3	4.47	0.64 (0.28,1.45)	0.28	2.87	0.74 (0.42,1.32)	0.31
SO_4^{2-}	µg/m^3	1.78	0.78 (0.35,1.72)	0.53	0.88	0.79 (0.49,1.27)	0.33
Black carbon	µg/m^3	0.23	0.91 (0.61, 1.36)	0.65	0.22	1.16 (0.79,1.69)	0.44
UFP	1000/m^3	4.26	1.82 (0.64, 5.15)	0.26	6.92	0.89 (0.52, 1.52)	0.66
O_3	ppb	6.69	1.01 (0.74,1.37)	0.95	6.74	0.77 (0.57, 1.04)	0.09
CO	ppm	0.11	1.13 (0.82,1.56)	0.46	0.13	1.20 (0.83,1.71)	0.33
NO	ppb	2.39	0.98 (0.85,1.14)	0.83	6.41	1.35 (0.98,1.85)	0.06
NO_2	ppb	2.52	1.03 (0.80, 1.33)	0.79	3.64	1.38 (0.98,1.93)	0.06

[a]From models adjusted for age, sex, race/ethnicity, visit, ambient and dew point temperature, barometric pressure, day of week, season and long-term temporal trends. Warm season was defined as April through September.
